# Tolerogenic and Activatory Plasmacytoid Dendritic Cells in Autoimmunity

**DOI:** 10.3389/fimmu.2013.00059

**Published:** 2013-03-06

**Authors:** Leslie Guéry, Stéphanie Hugues

**Affiliations:** ^1^Department of Pathology and Immunology, University of Geneva Medical SchoolGeneva, Switzerland

**Keywords:** plasmacytoid dendritic cells, type-I IFNs, antigen-presentation, tolerance, autoimmunity

## Abstract

Plasmacytoid dendritic cells (pDCs) are a particular subset of DCs that link innate and adaptive immunity. They are responsible for the substantial production of type 1 interferon (IFN-I) in response to viral RNA or DNA through activation of TLR7 and 9. Furthermore, pDCs present antigens (Ag) and induce naïve T cell differentiation. It has been demonstrated that pDCs can induce immunogenic T cell responses through differentiation of cytotoxic CD8^+^ T cells and effector CD4^+^ T cells. Conversely, pDCs exhibit strong tolerogenic functions by inducing CD8^+^ T cell deletion, CD4^+^ T cell anergy, and T_reg_ differentiation. However, since IFN-I produced by pDCs efficiently activates and recruits conventional DCs, B cells, T cells, and NK cells, pDCs also indirectly affect the nature and the amplitude of adaptive immune responses. As a consequence, the precise role of Ag-presenting functions of pDCs in adaptive immunity has been difficult to dissect *in vivo*. Additionally, different experimental procedures led to conflicting results regarding the outcome of T cell responses induced by pDCs. During the development of autoimmunity, pDCs have been shown to play both immunogenic and tolerogenic functions depending on disease, disease progression, and the experimental conditions. In this review, we will discuss the relative contribution of innate and adaptive pDC functions in modulating T cell responses, particularly during the development of autoimmunity.

## Plasmacytoid Dendritic Cell Characteristics

Plasmacytoid dendritic cells (pDCs) were first described as either interferon (IFN) producing cells (Ronnblom et al., 1983; Chehimi et al., 1989; Fitzgerald-Bocarsly, 1993) or as plasmacytoid monocytes or plasmacytoid T cells in reference to their plasma-like morphology in secondary lymphoid organs (SLOs) (Facchetti et al., 1988). They were further defined as pre-DC2, as during activation they can differentiate into conventional DCs (cDCs)-like cells. These cells exhibit a DC morphology, with increased MHC class II (MHCII) and costimulatory molecule expression, and the ability to induce naïve CD4^+^ T cell proliferation (Grouard et al., 1997). In 1999, the groups of M. Colonna and Y. J. Liu formally demonstrated that all these cell subtypes were actually the same entity, the pDCs (Cella et al., 1999; Siegal et al., 1999).

The development of pDCs occurs in the bone marrow (BM), after which they circulate through the blood stream, and reside in steady-state in the thymus and in the SLOs. Upon challenge via infection or inflammation, pDCs migrate and accumulate in inflamed tissues and draining lymph nodes (dLNs) (Reizis et al., 2011). The pDCs derive from the common dendritic progenitors (CDP) which express FLT3-R (CD135), CSF1-R (CD115), and low levels of c-kit (CD117) (Onai et al., 2007). Alternatively, pDCs may derive from lymphoid progenitors (Shigematsu et al., 2004; Luo and Lei, 2012; Sathe et al., 2013). Differentiation of pDCs relies essentially on Flt3-L which allows the expansion of cDC/pDC common progenitors and contributes to peripheral DC homeostasis (Waskow et al., 2008; Eidenschenk et al., 2010). The importance of E2-2 has been demonstrated for pDC differentiation (Cisse et al., 2008). It was further described that E2-2 drives the expression of other transcription factors involved in pDC fate, such as IRF-8 or Spi-B, while it inhibits other factors that are important for cDC differentiation, including Id-2 (Ghosh et al., 2010). Thus, a balance between the transcription factors E2-2 and Id-2 appears to control the differentiation toward the pDC lineage. Accordingly, in E2-2 deficient mice, pDCs exhibit an increased expression of Id-2 that correlates with a conversion into cDCs.

At the phenotypic level, pDCs are characterized by having intermediate (mouse) or no (human) expression of the DC marker CD11c. They are positive for the B cell marker CD45RA/B220, and express high levels of PDCA1, BST-2, Ly6C, and Ly49Q in mouse and BDCA-2, ILT-7, IL3Ra (CD123), and BDCA-4 in human (Reizis et al., 2011).

## Innate Plasmacytoid DC Functions

### Features of pDC innate functions

Plasmacytoid DCs are strong sensors of non-self nucleic acids derived from bacteria or viruses through binding to Toll-like receptors (TLR). Nucleic acids come from either viruses internalized by endocytosis, cytoplasmic viral RNA by autophagy (Lee et al., 2007), or other infected cells via exosome transport (Dreux et al., 2012). The receptors TLR7 and TLR9 are selectively expressed by pDCs. Interestingly, pDCs are the only DC subset expressing TLR9 in humans (Jarrossay et al., 2001; Kadowaki et al., 2001; Hornung et al., 2002) but not in mice (Chen et al., 2006). TLR7 senses guanosine or uridine rich single-stranded RNA from viruses or synthetic compounds such as imidazoquinoline and guanosine analogs (Diebold et al., 2004; Heil et al., 2004; Lund et al., 2004), while TLR9 recognizes single-stranded DNA containing unmethylated CpG motifs commonly found in viral and bacterial genomes (Hemmi et al., 2000; Bauer et al., 2001). The activation of TLR9 can also be through synthetic oligonucleotides (ODN) that mimic viral ssDNA responses (Kadowaki et al., 2001). TLR binding signals through MyD88, an adaptor protein that forms a signaling scaffold with IRAK-4, TRAF-6, and Btk, and induces the formation of the TRAF-3/IRAK-1/IKK-α/OPN/PI3K complex. As a consequence, IRF-7 is phosphorylated and subsequently translocated in the nucleus where it induces IFN-I gene transcription. Signaling through TRAF-6 also induces the NF-κB and MAPK pathways, leading to the secretion of inflammatory cytokines and chemokines, and the up-regulation of costimulatory molecules (Gilliet et al., 2008). Unlike other cells, where expression is dependent on IFNAR signaling, IRF-7 is constitutively expressed in pDCs (Honda et al., 2005b; Ito et al., 2006), possibly due to the low expression of the negative translational repressors 4EBPs (Colina et al., 2008).

The TLR ligands CpG-ODN have been classified as CpG-A, -B, and -C based on the different immune responses induced. In pDCs, CpG-A gives rise to robust IFN-I production, whereas CpG-B induces the production of inflammatory cytokines such as TNF or IL-6, as well as the up-regulation of MHCII and costimulatory molecules at the pDC surface (Kerkmann et al., 2005). These distinct effects of CpG-A and -B are pDC-specific and rely on different intracellular localizations after internalization. CpG-A possesses a poly-G tail, leading to the formation of a large multimeric complex that is retained in early endosomes and signals through MyD88 and IRF-7, thus inducing a strong IFN-I response. Conversely, monomeric CpG-B fails to be retained in early endosomes and rapidly travels to late endosomes/lysosomes to induce TNF and IL-6 production, and costimulatory molecule expression (Honda et al., 2005a; Guiducci et al., 2006).

Production of IFN-I by pDCs is much stronger (200–1000 times more effective) than any other cell type (Siegal et al., 1999). Multiple subtypes of IFN-I are secreted by pDCs, including IFNα, β, κ, ω, λ, and τ (Ito et al., 2006). During viral infections, pDC ablation selectively abrogates the early peak of IFN-I and leads to an increased viral burden (Swiecki et al., 2010), suggesting that IFN-I secretion is particularly crucial at the beginning of the antiviral response. By itself, IFN-I promotes the expression of IFN-stimulated genes that inhibit viral spreading through different mechanisms: (i) inhibition of viral replication by RNA degradation (Malathi et al., 2005) or by decreased protein synthesis (Barber et al., 1993); (ii) establishment of an antiviral state in uninfected cells; and (iii) induction of infected cell apoptosis (Pestka et al., 2004). Importantly, IFN-I links innate and adaptive immunity as it induces the differentiation, maturation, and activation of myeloid DCs that in turn promote antiviral T cell immunity (Paquette et al., 1998; Santini et al., 2000; Hibbert et al., 2003; Le Bon et al., 2003; Fonteneau et al., 2004; Yoneyama et al., 2005). Furthermore, IFN-I activates the antiviral functions of NK cells (Gerosa et al., 2005) and B cells (Jego et al., 2003). Using conditional targeting of the pDC-specific transcription factor E2-2, an elegant study has shown that pDC-deficient mice fail to clear chronic LCMV infection (Cervantes-Barragan et al., 2012). This defect correlates with impaired LCMV-specific CD4^+^ and CD8^+^ T cell numbers and functions, and relies on the lack of IFN-I production by pDCs, independently of their antigen (Ag) presenting capabilities.

### Innate pDC functions and autoimmunity

Self-DNA has been demonstrated to activate pDCs in autoimmune diseases. Under normal conditions, self-DNA is not recognized by pDCs since, when released by necrotic and apoptotic cells (Pisetsky and Fairhurst, 2007), it remains in the extracellular environment (Barton et al., 2006) and is rapidly degraded by DNAses. Following skin injury in psoriasis, self-DNA is released in the extracellular milieu (Lande et al., 2007) and sensed by pDCs (Nestle et al., 2005). In the wounded skin, the cationic anti-microbial cathelicidin LL37 is produced by keratinocytes and neutrophils (Zasloff, 2002). LL37 production is increased in psoriatic skin, binds the DNA released by dying cells, and forms aggregates which are resistant to extracellular nucleases (Lande et al., 2007). These complexes enter pDCs by endocytosis via lipid rafts and interactions with proteoglycans (Sandgren et al., 2004), and localize in early endosomes (similarly to CpG-A) to induce a strong IFN-I response. Consistently, an Imiquimod (TLR7 ligand)-containing topical cream was shown to exacerbate psoriatic lesions (Gilliet et al., 2004).

It has also been shown that IFN-I also plays a major role in the development of systemic lupus erythematosus (SLE) (Chan et al., 2012), by inducing the differentiation of both auto-antibody producing plasma cells (Jego et al., 2003; Thibault et al., 2009; Mathian et al., 2011) and cDC-driven effector T cells (Blanco et al., 2001). In SLE, apoptotic and necrotic cell derived self-DNA are complexed with (i) the peptide LL37, released by apoptotic neutrophil extracellular traps (NETs) in skin lesions (Bennett et al., 2003; Garcia-Romo et al., 2011; Lande et al., 2011), (ii) the protein HMGB1, also released in NETs, which binds aggregated nucleic acids (like CpG-A) (Tian et al., 2007), and (iii) by auto-antibodies directed against nucleic acids or nucleoproteins (Lovgren et al., 2004). These immune complexes allow the delivery of self-DNA into pDCs through the interaction of auto-Ab with FcγRII (CD32), and LC3-associated phagocytosis, a process described as a convergence between phagocytosis and non-conventional autophagy (Henault et al., 2012). In endosomes, self-DNA activates TLR signaling and subsequent IFN-I production (Lovgren et al., 2004; Barrat et al., 2005). Moreover, HMGB1 interacts with RAGE at the pDC surface, facilitating TLR9/DNA-binding in endosomes without inducing endocytosis (Tian et al., 2007). Finally, the resulting robust production of IFN-I, together with the presence of anti-LL37 auto-Abs in sera of SLE patients, induces the release of NETs and the continuous release of immune complexes (Garcia-Romo et al., 2011; Lande et al., 2011).

In multiple sclerosis (MS), many studies have suggested a protective role of pDCs and IFN-I. Indeed, relapsing patients treated with IFNβ-1a exhibit both a reduction in disease severity and a delay in relapses (Goodkin, 1996). In experimental autoimmune encephalomyelitis (EAE), a deficiency of IFNAR in central nervous system (CNS) myeloid cells exacerbates disease development (Prinz et al., 2008). In addition, pDCs have been shown to inhibit cDC functions and, consequently, dampen the development of encephalitogenic Th17 cells (Bailey et al., 2007), whereas anti-PDCA1 mediated pDC depletion increases EAE severity (Bailey-Bucktrout et al., 2008). As discussed by the authors, and consistent with the IFN-I dependent inhibition of Th17 inflammation in the CNS (Guo et al., 2008), pDC-mediated protection may indeed depend on IFN-I. However, the molecular mechanisms accounting for the local suppression of pathogenic T cells in the CNS by pro-inflammatory IFN-I remain unclear.

Until recently, the contribution of pDCs and IFN-I to pathogenesis in type 1 diabetes (T1D) remained controversial. The role of IFN-I, first believed to be protective (Sobel and Ahvazi, 1998; Sobel et al., 1998; Tanaka-Kataoka et al., 1999; Brod, 2002) has been revisited as IFN-I expression in insulin-producing β cells exacerbates T1D progression (Stewart et al., 1993; Alba et al., 2004). Furthermore, whereas pDCs were demonstrated to secrete IFN-I in dLNs from 3-week-old Non-Obese Diabetic (NOD) mice, pDC depletion in this strain dampened IFN-I production and diabetes progression. This protective effect is seemingly IFN-I dependent, since IFNAR blockade equally delayed diabetes onset (Li et al., 2008). Finally, Diana et al. recently formally demonstrated that IFN-α-producing pDCs are required for the initiation of diabetogenic T cell responses and T1D development. The study found that spontaneous β-cell death in young NOD mice induces the recruitment of B-1a cells, neutrophils, and pDCs in the pancreas. B-1a cells secrete anti-dsDNA IgGs which activate neutrophils that in turn release DNA-binding CRAMP (cathelicidin related anti-microbial peptide) in NETs. These immune complexes activate pDCs through TLR9, leading to local IFN-α production. Using depleting antibodies, the authors further demonstrated that IFN-α producing pDCs are essential to initiate T1D in NOD mice (Diana et al., 2013).

## Adaptive Plasmacytoid DC Functions

### pDCs function as bona fide Ag presenting cells

Accumulating evidence has revealed that pDCs can function as Ag presenting cells (APCs). In steady-state, pDCs can be easily detected in the blood, the thymus, and all SLOs (Bendriss-Vermare et al., 2001; Nakano et al., 2001; Summers et al., 2001; Asselin-Paturel et al., 2003; Seth et al., 2011). Upon inflammation, pDCs are recruited on a massive scale to infected or inflamed tissues, as well as to associated dLNs, and, importantly, in the LN T cell area, supporting a role for pDCs in activating naïve T cells (Cella et al., 1999; Krug et al., 2002; Vanbervliet et al., 2003; Irla et al., 2010). *In vitro*, pDCs exhibit the ability to capture, process, and present Ags through MHCI and MHCII molecules (Villadangos and Young, 2008; Tel et al., 2012). Steady-state pDCs were mainly described to be tolerogenic. Moreover, following TLR activation, pDCs upregulate MHCII and costimulatory molecules, which allow the direct modulation of the adaptive immune response. However, distinct mechanisms regulate Ag capture and processing in cDCs and pDCs, as well as resulting T cell outcome, suggesting complementary and, for the most part, non-overlapping functions of these two DC subsets.

Intracellularly Ag derived peptides either expressed by the cell itself (Krug et al., 2003; Young et al., 2008) or derived from intracellular-virus (Fonteneau et al., 2003; Salio et al., 2004; Schlecht et al., 2004; McGill et al., 2008; Young et al., 2008) are efficiently presented by pDCs through MHCI. Whether pDCs can phagocytose bacteria is still unclear (Villadangos and Young, 2008), while they have been shown to endocytose virions and exogenous proteins. pDCs express various endocytic receptors: Siglec-H (Zhang et al., 2006); the tetherin BST-2 (CD317) (Neil et al., 2008; Viswanathan et al., 2011); DCIR, which mediates clathrin-dependent endocytosis (Meyer-Wentrup et al., 2008); FcgRII (CD32), which allows the uptake of opsonized-Ags (Benitez-Ribas et al., 2006; Bjorck et al., 2008); and, specific to human pDCs, BDCA-2 which allows Ag delivery in the Ag processing compartment (Dzionek et al., 2001). Other receptors, such as ILT-7 (Cao et al., 2006) or NKp44 (Brown et al., 2004) may mediate endocytosis by pDCs, although this hypothesis needs further experimental confirmation (Villadangos and Young, 2008). Interestingly, ligation of these endocytic receptors potently suppresses IFN-I production by pDCs after TLR9 triggering (Dzionek et al., 2001; Blasius et al., 2004; Cao et al., 2007; Meyer-Wentrup et al., 2008). Finally, pDCs also acquire Ags from exosomes or apoptotic bodies (Hoeffel et al., 2007; Bastos-Amador et al., 2012).

Recent data suggests that mouse and human pDCs efficiently cross-present exogenous Ags to CD8^+^ T cells. *In vitro*, TLR-activated pDCs capture, process, and cross-present exogenous proteins to CD8^+^ T cells to induce proliferation, IFN-γ production, and cytolytic activity (Mouries et al., 2008; Kool et al., 2011). Accordingly, *in vivo* OVA delivery to CpG-activated pDCs via Siglec-H induces the generation of Ag-specific CD8^+^ T cells (Zhang et al., 2006), although T cell effector functions were not investigated in this study. More recently, it was shown that pDC-depleted Siglec-H-DTR mice immunized with OVA protein in the presence of TLR ligands exhibit impaired OVA-specific CD8^+^ T cell proliferation. Furthermore, the generation of MHC-I-OVA tetramer^+^ CD44^hi^ CD8^+^ T cells, as well as OVA-specific effector CTL functions, are impaired after pDC depletion (Takagi et al., 2011). However, this study cannot exclude that decreased CD8^+^ T cell activation and differentiation observed following pDC depletion does not reflect the abrogation of pDC cross-presenting functions, rather than simply the absence of pDC-mediated licensing of cDCs (Yoneyama et al., 2005). Accordingly, the depletion of cDCs in mice further co-immunized with OVA protein and CpG completely abrogates OVA-specific CD8^+^ T cell responses, suggesting that pDCs do not cross-present exogenous proteins to CD8^+^ T cells (Sapoznikov et al., 2007). Thus, the ability of murine pDCs to cross-present Ags to CD8^+^ T cells remains controversial. In humans however, it is accepted that blood pDCs efficiently cross-present viral Ags and initiate Ag-specific antiviral CD8^+^ T cell responses after being exposed to either influenza virus (Di Pucchio et al., 2008) or HIV-1 infected apoptotic cells (Hoeffel et al., 2007; Lui et al., 2009). The ability of pDCs to cross-present viral Ags does not seem to require IFN-I production but this function is strongly enhanced after TLR activation with synthetic compounds or influenza virus infection (Hoeffel et al., 2007).

Although some studies cited above suggested that the efficiency of Ag cross-presentation by pDCs to CD8^+^ T cells was comparable to that of cDCs, pDCs appear to be much less potent APCs compared to cDCs in stimulating CD4^+^ T cells. For instance, both *in vitro* and *in vivo*, CpG-activated pDCs were found not to be as efficient as cDCs at presenting exogenous Ag through MHCII to CD4^+^ T cells (Young et al., 2008; Kool et al., 2011). One possible explanation is that mouse cDCs and pDCs exhibit major differences in their Ag presentation machinery. Notably, MHCII molecules are differentially regulated in pDCs and cDCs. MHCII expression is regulated by the class II master transactivator gene CIITA, itself expressed under the control of distinct cell specific promoters (Reith et al., 2005). In particular, pDCs rely strictly on the B cell promoter pIII, whereas macrophages and cDCs depend on pI (LeibundGut-Landmann et al., 2004). In contrast to cDCs, activated pDCs fail to shut down MHCII synthesis (LeibundGut-Landmann et al., 2004) and turnover (Young et al., 2008), thereby allowing continued Ag presentation after activation. These features may indeed render pDCs better equipped for the continuous presentation of Ags, and allow them to present newly formed MHCII-peptide complexes even after activation (Sadaka et al., 2009), a feature with obvious advantages at sites of infection (Villadangos and Young, 2008). On the other hand, since pDCs lack the ability to accumulate long-lived MHCII-peptide complexes generated shortly after activation, it may also render these cells less efficient compared to cDCs at promoting effector CD4^+^ T cell responses, due to the low the exposure time of the peptide on cell surface.

### Impact on T helper functions

As discussed above, activated pDCs present Ags to naïve CD4^+^ T cells and thus directly contribute to T cell responses as APCs. In addition, pDCs can also indirectly impact T cells by producing inflammatory cytokines. As most studies eliminate pDCs genetically (Swiecki et al., 2010; Takagi et al., 2011) or through depleting mAbs (Bailey-Bucktrout et al., 2008; Jongbloed et al., 2009), it has been difficult so far to decipher the relative contribution of innate and adaptive pDC functions. However, accumulating evidence, mainly supported by *in vitro* studies, *in vivo* Ag targeting and abrogation of MHCII expression on pDCs, suggest a direct role of pDCs in impacting T cell responses. It is largely accepted that steady-state cDCs constantly present self- and non-self-Ags in a fashion that promotes T cell tolerance. Conversely, signals derived from pathogens or tissue damage generally boost cDC maturation, which promotes their capacity to induce effector T cell responses (Steinman, 2007). For pDCs however, their ability to promote either tolerance or immunity seems not to rely entirely on their activation state. Indeed, whereas immature pDCs exclusively promote tolerance, activated pDCs, depending on the anatomical localization and the cytokine milieu, may have both immunogenic and tolerogenic functions, although the exact nature of these functions remains to be established.

#### Immunogenic pDCs

**Figure 1:**
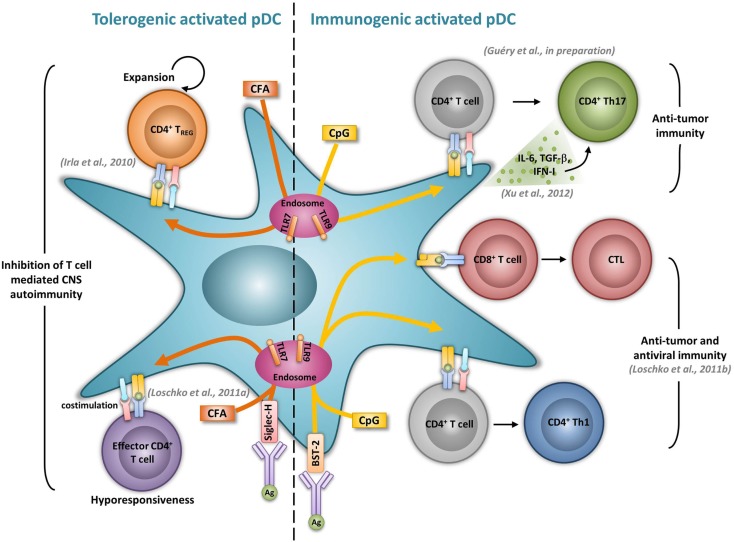
**Activated pDCs induce dual tolerogenic and immunogenic T cell responses**. The most relevant *in vivo* experimental animal systems are represented in this figure.

Previous studies performed *in vitro* showed that both human and mouse activated pDCs, given an antigenic peptide together with appropriate activating signals, activate naïve CD4^+^ T cells, and promote Th1 differentiation (Cella et al., 2000; Krug et al., 2001; Boonstra et al., 2003). In mice devoid of cDCs, CpG-treated LN pDCs promote Th1 development and memory differentiation *in vivo* (Sapoznikov et al., 2007). Similarly, Ag-specific delivery to TLR-activated pDCs via BST-2 induces Ag-specific Th1 development *in vivo*, as demonstrated by the production of IFN-γ by CD4^+^ T cells and subsequent immunoglobulin production (Loschko et al., 2011b) (Figure 1). pDCs have also been shown to induce Th17 responses in different experimental models. Human TLR7-triggered pDCs promote Th17 differentiation from either naïve or memory CD4^+^ T cells (Yu et al., 2010). In patients with GVHD, increased pDCs and Th17 cell numbers in the intestinal mucosa correlate with disease severity, suggesting a role of pDCs in driving Th17 responses during disease (Bossard et al., 2012). Furthermore, in tumor bearing mice, pDCs activation through CpG correlates with an increase of tumor-specific Th17 cells and an inhibition of the tumor growth (Xu et al., 2012) (Figure 1). The ability of pDCs to promote Th17 differentiation seems to be enhanced in the presence of TGF-β. Indeed, the transfer of TGF-β treated pDCs to collagen-induced arthritic mice leads to increased Th17 responses in LNs leading to increased disease severity (Bonnefoy et al., 2011). In EAE, anti-PDCA1 mediated pDC depletion resulted in impaired encephalitogenic Th17 responses significantly reducing early clinical scores (Isaksson et al., 2009). Moreover, pDCs may be able to convert T_regs_ into Th17 cells. Indeed, in rats, Foxp3^+^ T cells start to produce IL-17 when activated by mature pDCs (Gautreau et al., 2011). Using genetically modified mice selectively lacking MHCII expression by pDCs (LeibundGut-Landmann et al., 2004), we have found that CpG-activated pDCs function as APCs to induce Th17 responses *in vivo* (Guery et al., manuscript in preparation) (Figure 1). Our unpublished data further suggest that the ability of activated pDCs to promote Th17 cells may be used as a vaccination strategy against tumors.

#### Tolerogenic pDCs

Plasmacytoid DCs have also been demonstrated to be involved in the induction of central and peripheral tolerance. It has been suggested that the function of pDCs within the thymus, as in other tissues, might simply be to protect the tissue from viral infections (Wu and Shortman, 2005). However, it was recently suggested that recirculating pDCs might present self-Ags in the thymus and contribute to the inactivation, or deletion, of autoreactive T cells. pDCs were detected in human thymus, colocalize with Foxp3^+^ T_regs_, and, when activated with CD40L plus IL-3, efficiently promote the development of Foxp3^+^ natural T_regs_ (nT_regs_) from autologous thymocytes (Martin-Gayo et al., 2010). Similarly, human thymic pDCs activated with CpG and TSLP induce nT_reg_ generation (Hanabuchi et al., 2010). In these two studies, T_regs_ generated by pDCs produce more IL-10 and less TGF-β compared to nT_regs_ primed by cDCs under the same conditions, suggesting a complementary effect of the two DC subsets in the development of central tolerance. However, whether pDCs actually promote thymocyte differentiation into nT_reg_ *in vivo* remains to be firmly demonstrated. In contrast to human pDCs, murine thymic pDCs do not efficiently induce T_reg_ differentiation from thymocytes *in vitro* (Proietto et al., 2009). *In vivo*, a recent study illustrated the importance of CCR9 in targeting peripheral immature pDCs to the thymus. This indicates a role for pDCs in presenting extrathymically acquired Ags to further induce the deletion of Ag-specific CD4^+^ thymocytes (Hadeiba et al., 2012). No role for pDCs in the generation of nT_regs_ was observed in this study, suggesting that mouse pDCs are intrinsically inefficient at inducing T_regs_ in the thymic environment *in vivo*.

**Figure 2:**
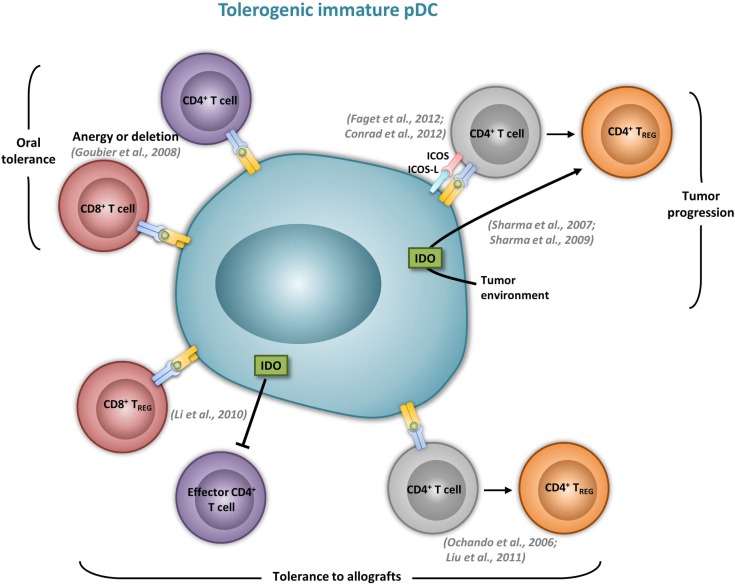
**Immature pDCs induce tolergenic T cell responses**. The most relevant *in vivo* experimental animal systems are represented in this figure.

Although it was suggested that pDCs are poor presenters of Ag in the absence of microbial stimulation (Colonna et al., 2004), immature pDCs promote T cell anergy, T cell deletion, as well as T_reg_ differentiation. In humans, freshly isolated pDCs induce anergy in human CD4^+^ T cell clones, but it is reversed upon the addition of exogenous IL-2 (Kuwana, 2002). In mice, pDCs prevent oral T cell priming and are responsible for systemic tolerance to dietary Ags including proteins and haptens (Goubier et al., 2008). Indeed, pDC depletion induces hypersensitivity and CD8^+^ T cell responses toward oral Ags. In contrast, the transfer of Ag-loaded immature pDCs in naive mice suppresses both Ag-specific CD4^+^ and CD8^+^ T cell responses by inducing either anergy or deletion, suggesting that oral tolerance relies on Ag presentation by pDCs (Goubier et al., 2008) (Figure 2). Immature pDCs expressing CCR9 were defined as inducers of Foxp3^+^ T_regs_ that suppress Ag-specific immune response in a GVHD model. Importantly, transferred CCR9^+^ pDCs efficiently suppressed allogenic GVDH (Hadeiba et al., 2008). pDCs were also identified as phagocytic APCs essential for tolerance to vascularized cardiac allografts (Ochando et al., 2006) (Figure 2). In this model, alloantigen-presenting pDCs home to the LNs under tolerogenic conditions, where they mediate alloantigen-specific T_reg_ development and allograft tolerance. In an other model of cardiac allograft transplantation, the absence of pDCs in LNs from CCR7^−/−^ mice impairs T_reg_ induction, and results in graft rejection, whereas pDC transfer restores both T_reg_ frequencies and tolerance to the cardiac allograft (Liu et al., 2011) (Figure 2). In rats, pDCs also induce tolerance to allografts by inhibiting CD4^+^ T cells either directly through an indoleamine 2,3-dioxygenase (IDO)-dependent mechanism, or indirectly through the induction of CD8^+^ T_regs_ (Li et al., 2010) (Figure 2). Furthermore, pDC depletion in mice induces classical features of asthma after inhalation of an inert Ag, including IgE sensitization, airway eosinophilia, goblet cell hyperplasia, and Th2 cytokine production. In contrast, the adoptive transfer of pDCs before sensitization prevents asthma, possibly through the induction of T_regs_ (de Heer et al., 2004). Using Siglec-H inducible deficient mice, a recent study nicely showed that Siglec-H controls the ability of steady-state pDCs to induce the conversion of naïve CD4^+^ T cells in inducible T_regs_ (iT_regs_) *in vivo* (Takagi et al., 2011). In the contexts of tumors, the microenvironment maintains a resting pDC phenotype, characterized by low expression of costimulatory molecules and low IFN-I production (Hartmann et al., 2003; Conrad et al., 2012; Sisirak et al., 2012). Consequently, pDCs induce mainly tolerogenic tumor CD4^+^ T cell responses, through IDO-dependent T_reg_ generation (Sharma et al., 2007) (Figure 2). Accordingly, IDO inhibition in pDCs promotes the conversion of T_regs_ into Th17 cells that efficiently inhibit tumor growth (Sharma et al., 2009). ICOS-L expression by pDCs in the tumor context has also been implicated in their ability to generate T_regs_ (Conrad et al., 2012; Faget et al., 2012) (Figure 2).

Activated pDCs were demonstrated to favor T_reg_ development. T_reg_ induction has been correlated with low peptide-MHCII densities on APCs (Kang et al., 2007; Turner et al., 2009). Thus, the ability of activated pDCs to induce T_regs_ might be explained by the fact that they do not stabilize peptide-MHCII complexes at their cell surface, and, thus, provide a weak TCR engagement promoting T_reg_ development. In humans, TLR-activated pDCs induce the development of IL-10 producing T_regs_ in an ICOS dependent manner (Ito et al., 2007; Ogata et al., 2013). Furthermore, HIV-stimulated IDO expressing pDCs induce the differentiation of naive CD4^+^ T cells into T_regs_ (Manches et al., 2008). CpG-activated human pDCs can also promote T_reg_ differentiation (Moseman et al., 2004), possibly in an IDO-dependent fashion (Chen et al., 2008). Finally, TLR-activated rat pDCs induce T_reg_ proliferation *in vitro* (Ouabed et al., 2008).

The severity of several autoimmune diseases has been demonstrated to be regulated by pDCs. In rheumatoid arthritis, mature pDCs from patients express high levels of IDO and are necessary for the differentiation of allogeneic naïve CD4^+^ CD25^−^ T cells into IL-10 producing T_reg_ (Tr1) cells (Kavousanaki et al., 2010). Moreover, in an *in vivo* mouse model of arthritis, pDC depletion correlates with enhanced articular pathology and increased T and B cell autoimmune responses to type II collagen (Jongbloed et al., 2009). In lupus, low dose Ag therapy induces the production of TGF-β by pDCs and dampens their ability to respond to TLR stimulation. The transfer of these tolerogenic pDCs promotes T_reg_ expansion while simultaneously suppressing inflammatory Th17 infiltrating the kidney of lupus-prone mice (Kang et al., 2007). Ag targeting in pDCs has also been shown to inhibit T helper cell dependent autoimmunity. In EAE, Siglec-H mediated MOG_35–55_ delivery to pDCs dampens EAE, by inducing MOG-specific CD4^+^ T cell hyporesponsiveness resulting in the impaired induction of Th1 and Th17 cells, without promoting T_reg_ differentiation (Loschko et al., 2011a) (Figure 1). Using a genetic mouse model in which MHCII is specifically abrogated in pDCs, we also identified a tolerogenic role for Ag presenting pDC functions during EAE. We demonstrated that pDCs, by presenting myelin Ags to naïve CD4^+^ T cells, induce the expansion of nT_regs_. As a consequence, mice carrying a selective abrogation of MHCII on pDCs exhibit impaired nT_reg_ expansion, increased encephalitogenic Th1 and Th17 responses and exacerbated EAE (Irla et al., 2010) (Figure 1).

As discussed before, pDCs contribute to the pathology of T1D through the production of IFN-I. However, pDCs are also implicated in the inhibition of diabetogenic T cells during infections. In RIP-LCMV mice, OX40-OX40L dependent pDC–iNKT cell interactions control viral replication in pancreatic islets of LCMV infected mice by inducing IFN-I production by pDCs (Diana et al., 2009). In addition, this pDC–iNKT cooperation has been reported to promote TGF-β production by pDCs, that in turn, acquire the ability to convert naive anti-islet CD4^+^ T cells into Foxp3^+^ T_regs_ in pancreatic dLNs. These T_regs_ are then recruited in the pancreatic islets where they produce TGF-β that inhibits islet-specific CD8^+^ T cells and dampens T1D severity (Diana et al., 2011). In NOD mice, pDC depletion leads to accelerated insulitis and disease onset, and correlates with a local loss of IDO, suggesting that during T1D, pDCs may exert IDO-dependent tolerogenic functions by regulating islet-specific CD4^+^ T cell responses (Saxena et al., 2007).

In summary, pDCs in SLO were shown to contribute to T cell tolerance in several experimental systems, regardless of whether they exhibit a steady-state or activated phenotype (Figures 1 and 2). Interestingly, tolerogenic pDC functions were demonstrated to be dependent on IDO in several models. Furthermore, IDO contribution does not seem to rely on a pDC inflammatory environment. Why and how IDO is involved in the ability of pDCs to dampen some, but not all, Ag-specific T cell responses is still a matter of debate. This phenomenon is more complex due to recent findings demonstrating a second function for pDC-derived IDO. Together with the tryptophan catalytic activity inhibiting effector T cell function, IDO has been postulated to act as a signaling protein in response to TGF-β inducing the conversion of naïve CD4^+^ T cells into T_regs_ (Pallotta et al., 2011). Thus, the relative contribution of the dual IDO functions needs to be addressed in the different models where an IDO-dependent inhibition of T cell responses by pDCs has been described.

## Concluding Remarks

Thus, steady-state pDCs exclusively promote T cell tolerance. However, the emerging picture of pDC functions during the development of inflammatory autoimmune disorders is that they contribute to disease pathogenesis by the production of IFN-I, while promoting self-Ag-specific CD4^+^ T cell tolerance though their ability to present auto-Ag. Altogether, studies show that innate and adaptive pDC functions may have opposite effects on T cell tolerance toward self-tissues. Interestingly, the engagement of endocytic receptors favors Ag presenting pDC functions, while it dampens their ability to produce IFN-I, suggesting that Ag targeting in pDCs would represent an attractive therapeutical strategy to control autoimmunity and graft rejection.

## Conflict of Interest Statement

The authors declare that the research was conducted in the absence of any commercial or financial relationships that could be construed as a potential conflict of interest.
